# Effect of high proportion concentrate dietary on Yak jejunal structure, physiological function and protein composition during cold season

**DOI:** 10.1038/s41598-021-84991-3

**Published:** 2021-03-09

**Authors:** Jianlei Jia, Chunnian Liang, Xiaoyun Wu, Lin Xiong, Pengjia Bao, Qian Chen, Ping Yan

**Affiliations:** 1 Key Laboratory of Yak Breeding Engineering of Gansu Province, Lanzhou Institute of Husbandry and Pharmaceutical Sciences, Chinese Academy of Agricultural Sciences, Lanzhou, 730050 China; 2 State Key Laboratory of Plateau Ecology and Agriculture, Qinghai University, Xining, 810016 China

**Keywords:** Animal physiology, Mass spectrometry

## Abstract

The current study aimed to investigate the damage of long-term high concentrate diet feeding pattern on Yak jejunal structure, physiological function and protein composition during cold season. Twelve Datong male Yak (*Bos grunniens*) with the same age from cold season were randomly selected and slaughtered to determine Yak jejunal digestive enzyme activity, morphology and protein composition on different feeding patterns in Tibetan Plateau. The results showed that Yak jejunum digestive enzyme activity and morphology of grazing reared group were better than those in the intensively reared group. A total of 96 differentially expressed proteins were identified by label-free Mass Spectrometry (MS), which could be concluded to two predominant themes: protein structure and inflammatory response. Nine differentially expressed proteins were correlated in Yak jejunum damage in different feeding patterns. According to this research, we found that feeding pattern resulted the differences in Yak jejunum physiological function, morphology and protein composition. This fact was confirmed long-term high dietary concentrate feeding could damage the jejunum epithelial morphology and function.

## Introduction

Yaks (*Bos grunniens*) live mainly above an altitude of 3000 m on the Tibetan Plateau and have developed an ability to survive and reproduce under harsh conditions during long periods of natural selection and artificial domestication^1^. Yak production is an economic pillar of the Tibetan Plateau area. The Tibetan Plateau is known for its extremely harsh conditions, characterized by high altitude, severe cold, low atmospheric oxygen, strong ultraviolet radiation and short forage growing season. Herbage and nutrients are insufficient to support livestock during the cold period, especially those raised under traditional grazing management^2^.

The modern intensive management pattern has gradually replaced traditional grazing patterns in the Tibet plateau area^3^. This pattern concentrates the diet by more than 75%, leading to accelerated daily bodyweight gain in yaks and increased economic benefits for herdsman. Ruminants must consume a diet with 40–70% roughage to maintain normal rumen function and the gastrointestinal microflora environment^4^. However, long-term feeding of a highly concentrated diet can eventually have severe impacts on animal health, leading to metabolic dysfunction related to rumen microorganisms, such as accumulation of volatile fatty acids (VFAs) and decreases in pH. Fermentable carbohydrates enter the small intestine through the rumen, causing intestinal acidosis, changes in the microbial community structure and destruction of intestinal epithelial morphology and structure^5^.

Dietary nutrients are digested in the intestine by digestive enzymes and absorbed through the intestinal epithelium to provide energy for animals^6^. The jejunum is an important organ for nutrient digestion, absorption and metabolism and acts as a barrier against harmful substances. It also plays an important role in yak health. Endogenous jejunal barriers can be damaged by external factors, leading to enteric flora disturbance and decreased production performance. Under traditional grazing management, yaks can graze on pastures without dietary supplementation throughout the year, and a unique intestinal environment and digestive function develop to enable the yaks to adapt to plateau grazing conditions. However, the modern intensive management pattern changes yak dietary habits; feeding of diets with high proportions of concentrate leads to reduced feedstuff residence time in the yak rumen and increases the levels of carbohydrates and microbial fermentation in the jejunum, which negatively affects the monolayer structure of jejunum epithelial cells and damages the intestinal barrier^7,8^. This may be an important physiological mechanism by which highly concentrated diets destroy the Yak jejunal structure and barrier function. Here, we explored jejunal barrier dysfunction caused by long-term high concentrate diet feeding during the cold season. Twelve Datong male yaks with different feeding patterns (intensive and grazing) were randomly selected and slaughtered during the cold season. Jejunal digestive enzyme activity was determined by ELISA, and jejunal morphology was investigated with frozen sections. A label-free method was used to identify proteins that were specifically expressed in yaks with different feeding patterns, and we analyzed the signaling pathways of selected proteins. Collectively, our results shed new light on the damage caused by long-term highly concentrated diet feeding on the yak jejunum. In addition, this study lays a technical foundation for understanding the effects of the intensive rearing pattern.

## Results

### Analysis of digestive enzyme activity in the jejunum

The activity levels of amylase (AMS), lipase (LPS), trypsin (TRS) and carboxymethyl cellulase (CLS) in yaks with different feeding patterns are presented in in Table 1. The data indicated that AMS and CLS activity was higher in the grazing-reared group than in the intensively reared group, but the difference between the two groups was not significant (*P* > 0.05). The LPS activity of the intensively reared group was significantly greater than that of the grazing-reared group (*P* < 0.05). In contrast, the TRS activity of the intensively reared group was significantly lower than that of the grazing-reared group (*P* < 0.05). Overall, Yak jejunal digestive enzyme activity was higher in yaks subjected to the grazing rearing pattern than in yaks subjected to the intensive rearing pattern.

**Table 1:** The alkaline phosphatase activity of yak jejunum in different feeding pattern.

	AMS	LPS	CLS	TRS
Grazing group	0.176 ± 0.043	0.218 ± 0.072^a^	4.102 ± 0.835	70.642 ± 7.206^a^
Intensively group	0.121 ± 0.071	0.542 ± 0.053^b^	2.145 ± 0.332	36.109 ± 3.058^b^

Different letters represent significant differences (P < 0.05).

### Histological analysis of jejunal morphology

The villus height, villus width, crypt depth, mucosal thickness and muscular thickness of the yaks in the grazing-reared and intensively reared groups are summarized in Table 2. The villus height, villus width, mucosal thickness and muscular thickness of the jejunum were significantly greater in the grazing-reared group than in the intensively reared group (*P* < 0.05). However, the crypt depth was smaller in the grazing-reared group than in the intensively reared group, although the difference was not significant (*P* > 0.05).

**Table 2:** The villous height, villous width, crypt depth, mucosa thickness and muscular thickness of yak jejunum in different feeding pattern.

	Villous height	Villous width	Crypt depth	Mucosa thickness	Muscular thickness
Grazing group	563.33 ± 11.48^a^	149.29 ± 2.67^a^	178.84 ± 6.59	808.38 ± 15.52^a^	528.91 ± 20.93^a^
Intensively group	408.31 ± 10.46^b^	82.88 ± 5.45^b^	194.61 ± 8.37	939.18 ± 14.14^b^	751.51 ± 15.14^b^

Different letters represent significant differences (P < 0.05).

### Differences in proteome profiles between the different feeding patterns

**Figure 1:**
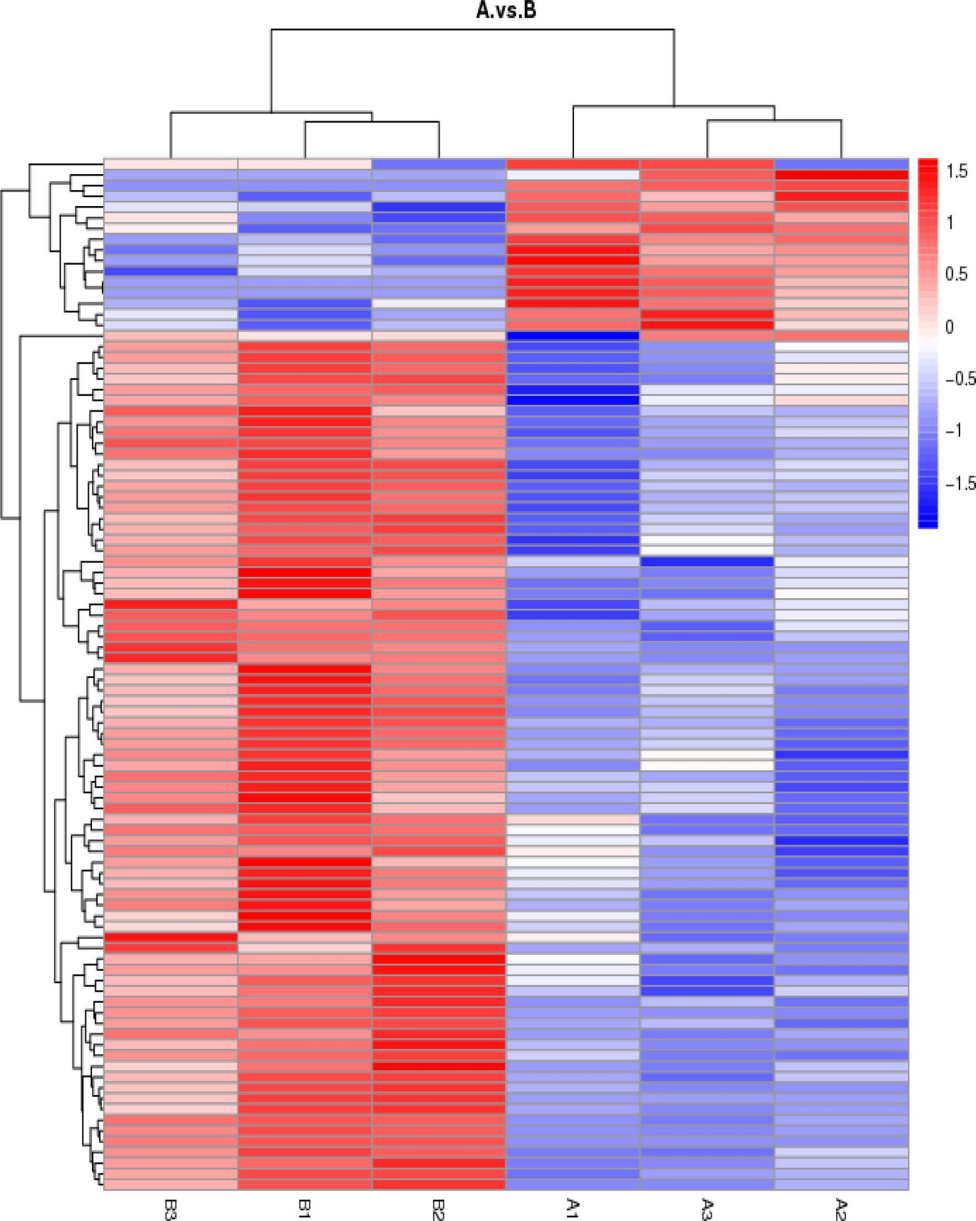
Differentially expressed proteins, including 3 replicates with yak jejunum during grazing reared group (**A**) and intensively reared group (**B**). The image presents the relative abundance of proteins using different colors, where deeper red represents higher intensity and blue represents lower intensity.

We successfully identified 1208 proteins with the Bos taurus reference database using Mascot and a label-free method. Then, we applied a manual thresholding approach and a probabilistic prediction algorithm to obtain 1066 high-confidence candidates. Of these 1066 proteins, 96 differentially expressed proteins were identified between the intensively reared group and grazing reared group using a threshold of a P < 0.05 or a fold change of > 1.2 or < 0.83 (Table S1). There were 17 upregulated and 79 downregulated differentially expressed proteins in the grazing-reared group compared to the intensively reared group (Fig. 1).

### Bioinformatics analyses

**Figure 2:**
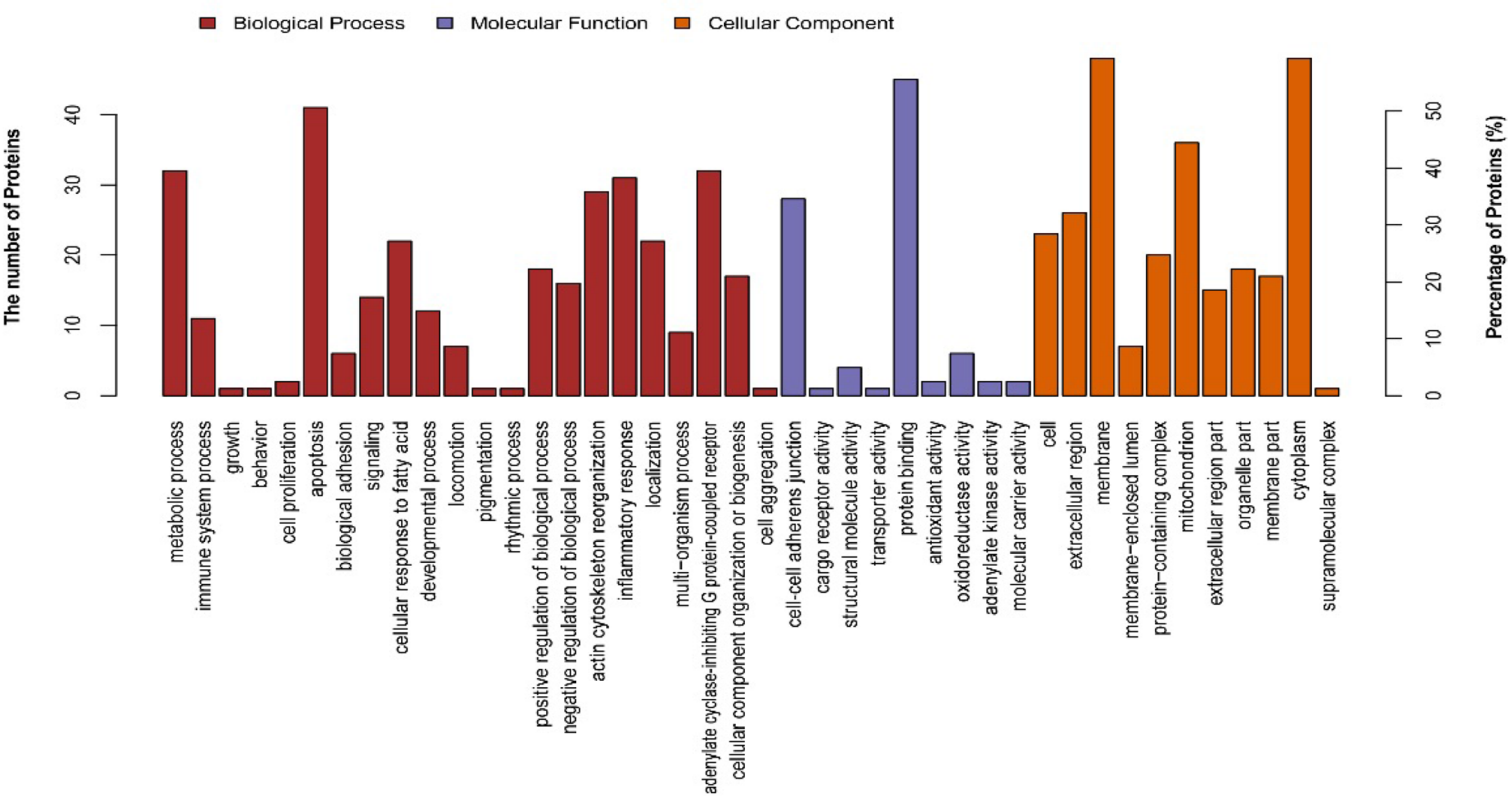
GO analysis of differentially expressed proteins.

To explore the biological functions associated with the differentially expressed proteins in the jejuna of yaks subjected to different feeding patterns, we performed Gene Ontology (GO) enrichment analysis and annotated the proteins with cellular component (CC), molecular function (MF) and biological process (BP) terms (Fig. 2). The most highly enriched terms associated with the differentially expressed proteins in the CC category were the membrane (GO:0016020), cytoplasm (GO:0005737) and mitochondrion (GO:0005739; GO:0005740) terms. Other enriched terms in the MF category were the oxidoreductase activity (GO:0016491), cell–cell adherens junction (GO:0005525), adenylate kinase activity (GO:0004017), protein binding (GO:0019904) and structural molecule activity (GO:0003954) terms. The apoptosis (GO:0048468), inflammatory response (GO:0016020), cellular response to fatty acid (GO:0008152), actin cytoskeleton reorganization (GO:0055114) and adenylate cyclase-inhibiting G protein-coupled receptor (GO:0046034) terms were enriched in the BP category.

**Figure 3:**
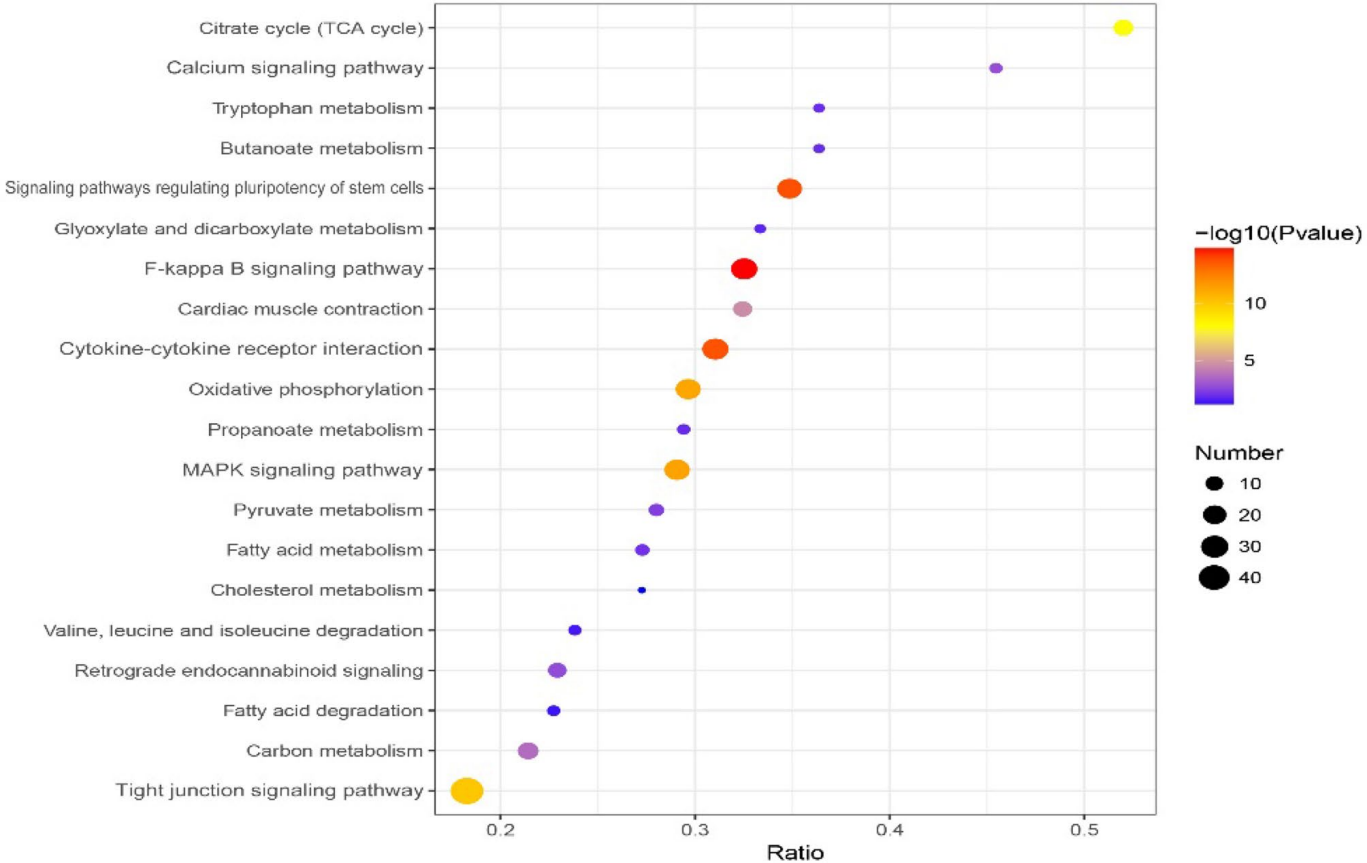
KEGG analysis of differentially expressed proteins (Fisher’s exact test, P value < 0.01).

The major pathways associated with the differentially expressed proteins in the yak jejunum between the grazing-reared group and the intensively reared group were identified via Kyoto Encyclopedia of Genes and Genomes (KEGG) pathway analysis using KEGG Automatic Annotation Server (KAAS) software. The KEGG results showed that out of a total of 71 pathways, 49 were significantly enriched (P < 0.05). The top 6 of 20 significantly enriched pathway terms, ranked by significance and percent overlap, were the tight junction signaling pathway (ko04530), NF-kappa B signaling pathway (ko04064), MAPK signaling pathway (ko04010), cytokine–cytokine receptor interaction (ko04060), calcium signaling pathway (ko04020) and signaling pathways regulating pluripotency of stem cells (ko04550, Fig. 3) terms. The results of the KEGG pathway analysis matched the results of the GO analysis.

**Figure 4:**
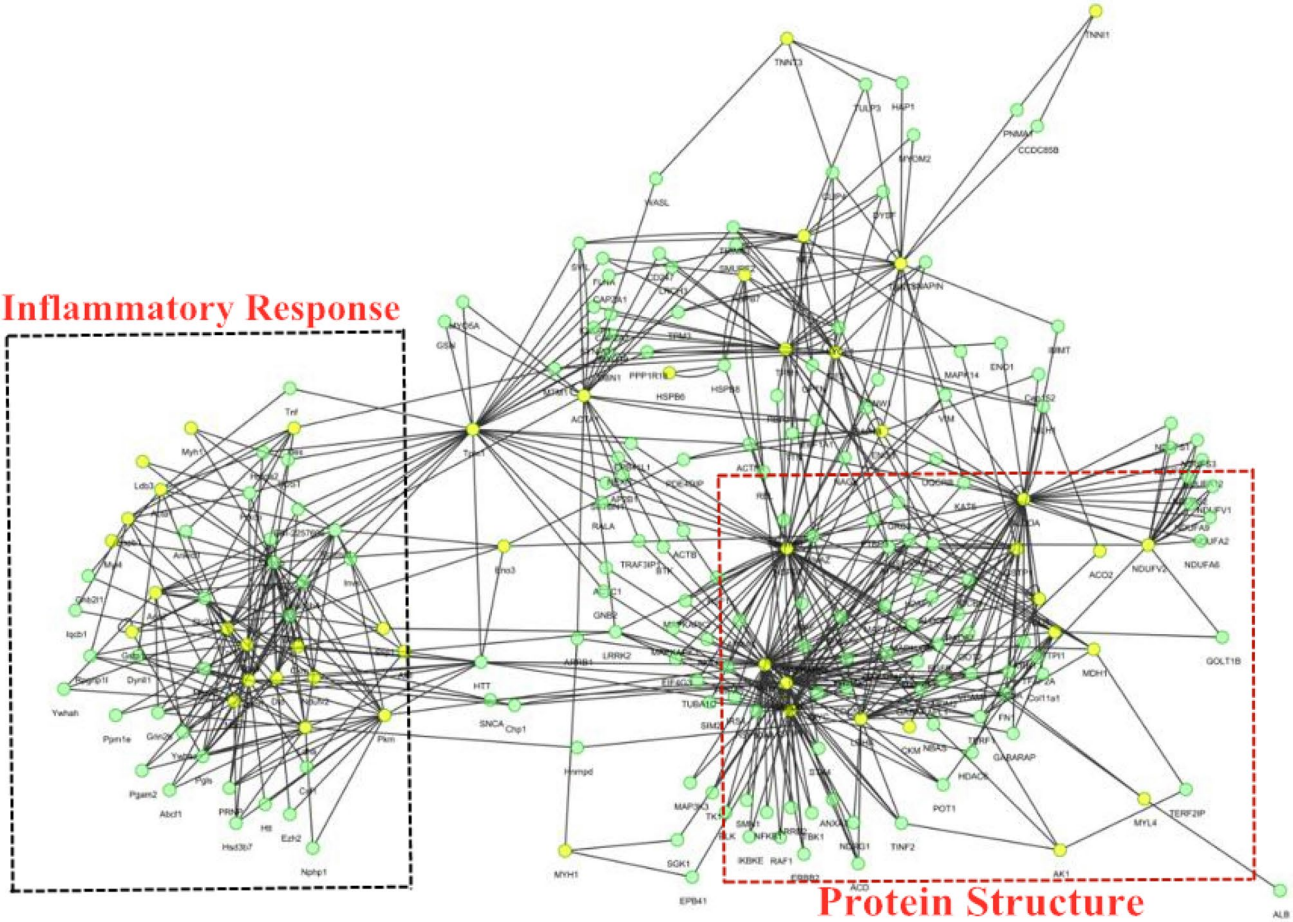
PPIs analysis of differentially expressed proteins. Protein–protein interaction networks of the differential abundance proteins of different feeding groups in Yak jejunum based on Cytoscape software. The nodes were proteins from Bos taurus database and the lines were the predicted functional annotations.

To gain information on the interaction network, possible interactions among the 96 proteins were analyzed online using the UniProt database. As expected, the 96 target proteins constituted a complex and strong protein–protein interaction (PPI) network, and the results of this analysis identified two predominant themes: protein structure and the inflammatory response. To provide further insights into the biological processes identified by this approach, we used Fisher's test (the A/B significance test) for target terms. Target terms were defined as the terms in the BP category that were enriched for the most differentially abundant proteins in our results, such as the tight junction term (Occludin, Claudin, Zo-1, ATP1B3 and HKDC1) and the inflammatory reaction term (GPR41, IL-6, TNF-α and CCL5) (Fig. 4).

Validation of the expression of differentially expressed protein-coding genes by quantitative real-time PCR (RT-PCR).

**Figure 5:**
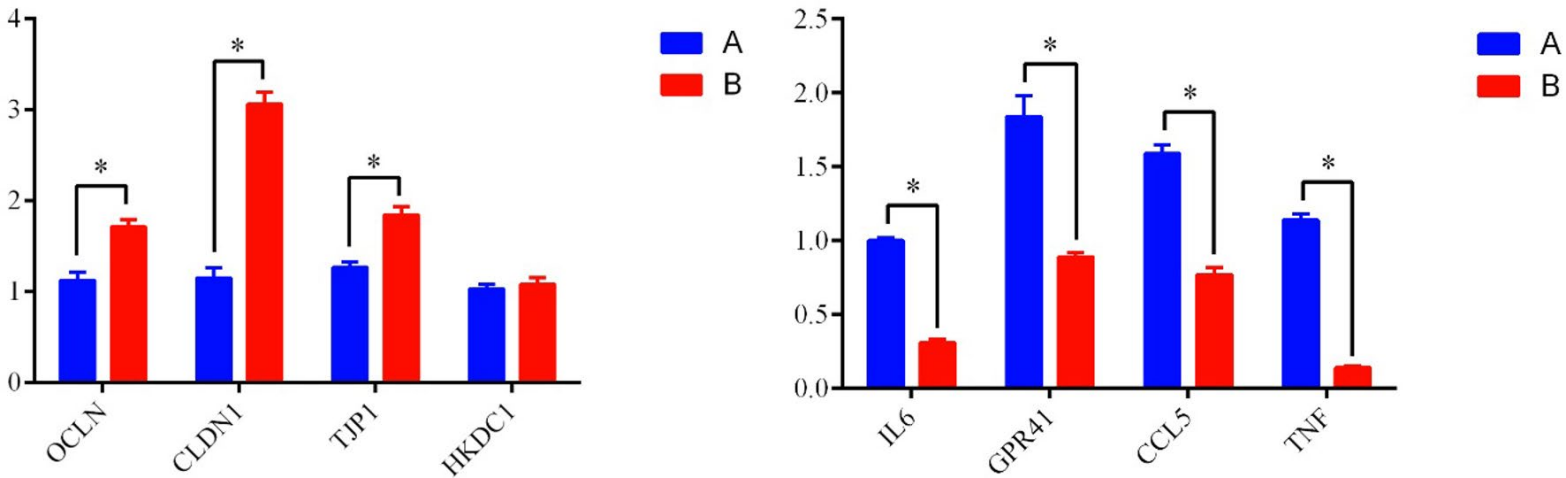
Effects of different feeding pattern on the differentially expressed proteins coding genes in yak jejunum during intensively reared group (**A**) and grazing reared group (**B**).

As shown in Fig. 5, the gene expression levels of OCLN, CLDN1, TJP1 and HKDC1 were determined to be higher in the grazing-reared group than in the intensively reared group (*P* < 0.05) via RT-PCR, but ATP1B3 expression was not significantly different between the two groups. In addition, the gene expression levels of IL6, GPR41, CCL5 and TNF were higher in the intensively reared group than in the grazing-reared group (*P* < 0.05). In summary, RT-PCR revealed that the genes encoding the selected proteins determined to be differentially expressed by label-free analysis exhibited the same expression tendencies as the proteins. The RT-PCR assay thus illustrated that the label-free proteomics results were reliable for further analyses.

## Discussion

The jejunum is the main organ for dietary nutrient digestion, absorption and metabolism and plays an important role in yak health. Under the modern intensive management pattern, the diet is concentrated by more than 75% to enhance daily gain and performance. However, long-term highly concentrated diet feeding severely impacts animal health; for example, it causes jejunal acidosis^4^. The levels of dietary nutrient digestion, absorption and metabolism can be reflected by digestive enzyme activity. Starches are the main energy sources for ruminants. AMS can hydrolyze starch to glucose, maltose and oligosaccharides by acting on the 4-c-d-glycosidic bonds of the starch molecule^13^. Fat is a necessary material for energy storage and nutrition in animals, and essential fatty acids are provided mainly by dietary fat. LPS can digest dietary fat into absorbable nutrients, such as free fatty acids, glycerin and monoglycerides. In addition, TRS can hydrolyze dietary proteins into smaller molecules, amino acids; amino acids are involved in intracellular signaling transmission and apoptosis and play vital roles in animal immune responses^14^. Ruminant cellulose degradation depends primarily on cellulase secreted by microorganisms, and CLS is an important enzyme for cellulose degradation^15^. In our study, Yak jejunal digestive enzyme activity was weakened with long-term highly concentrated diet feeding. We observed that the digestion and absorption of the grazing-reared group were better than those of the intensively reared group.

Morphophysiological variations in the jejunal epithelia of ruminants can be used to identify variations in dietary nutrient digestion and absorption^11^. Characteristics of the villus absorptive surface (height, width and crypt depth) in the jejunal epithelium are closely related to the extent of enterocyte development and are thus direct representations of the quality of the intestinal environment and indicators of intestinal health^16^. The present study showed that dietary nutrient digestion and absorption ability increased with increasing villus absorptive surface area and that the submucosal and muscular thickness of the jejunum decreased with long-term highly concentrated diet feeding, decreasing the absorption of nutrients in the yak jejunum. Long-term highly concentrated diet feeding caused many nutrients to be degraded into short-chain fatty acids (SCFAs) by microorganisms in the rumen, leading to an increase in the jejunal VFA content and a decrease in the jejunal pH value. The VFAs had high lipid solubility when the pH value dropped below a critical value. VFAs can infiltrate into jejunal mucosa cells to acidify cells, damaging the intestinal barrier and causing an inflammatory response^17,18^. We found that various jejunal characteristics were significantly decreased in the intensively reared group of yaks, such as the villus height, villus width, mucosal thickness and muscular thickness. These results are consistent with those of previous studies in ruminants.

SCFAs in the rumen permeate into the yak jejunum and destroy the structural integrity of the jejunum, causing morphophysiological damage and epithelial barrier dysfunction^19^. The mechanical barrier formed by the normal morphological structure of the mucosa plays a protective role in the jejunum^20–22^. Tight junction proteins are important parts of the jejunal mechanical barrier and include transporter proteins such as occludin and claudin, which are involved mainly in enterocyte proliferation, differentiation, apoptosis and cell membrane permeability regulation. Mechanical barrier damage-induced jejunal dysfunction allows pathogens to infect enterocytes, which results in tight junction structural damage, increased permeability and an inflammatory response^19^. According to recent reports and our study, the target proteins are associated with two predominant themes: protein structure (the tight junction signaling pathway, the calcium signaling pathway and signaling pathways regulating the pluripotency of stem cells) and the inflammatory response (the MAPK signaling pathway, the chemokine signaling pathway and cytokine–cytokine receptor interactions). GPR 41, a key activator of the jejunal mechanical barrier, participates in regulating the inflammatory response and affects tight junction signaling pathways^23^. Consumption of a highly concentrated diet can initiate the enterocyte gluconeogenesis process, activate the tight junction signaling pathway and regulate related downstream apoptosis pathways, decreasing the expression of tight junction proteins, such as occludin, claudin and Zo-1. In addition, proliferation of regulatory T cells promotes inflammatory response (MAPK) pathway-related gene expression, inducing the secretion of many anti-inflammatory cytokines (IL-6, TNF-α and CCL5)^24–26^. GPR41 was detected in the current study. Long-term highly concentrated diet feeding changed the normal jejunal metabolic pathways. Several identified candidate proteins were identified by label-free MS and were confirmed by RT-PCR, including occludin, claudin, Zo-1, ATP1B3, HKDC1, IL-6, TNF-α and CCL5. Compared with the grazing-reared group, the intensively reared group exhibited significantly lower jejunal expression levels of occludin, claudin, Zo-1, ATP1B3 and HKDC1. However, the opposite tendency was observed for IL-6, TNF-α and CCL5, indicating that the large amounts of SCFAs in the jejuna of the yaks fed a highly concentrated diet could damage the mechanical barrier and reduce the expression of proteins. In addition, GPR41 was activated to upregulate the expression of proinflammatory factors. In summary, the results provide evidence that highly concentrated diet feeding damages the yak jejunum.

## Material and methods

### Study site

The study was performed in Haibei Tibetan Autonomous Prefecture, Qinghai Province, China, which is situated on the northeastern Qinghai–Tibetan Plateau. This area is over 3000 m above sea level and has a dry, cold climate.

### Animals and diets

Twelve Datong male yaks (4 ± 0.5 years old) that grazed on natural grasslands in Haibei Tibetan Autonomous Prefecture were selected. All the yaks were distributed into two groups. The yaks of the experimental group were fed in a roofed shed via an intensive rearing pattern, and the yaks of the control group were allowed to graze in a pasture during the experimental period. The experiment lasted for 180 days from 16 November 2018 to 4 May 2019. The first 10 days were used as an adjustment period for the experimental group. The following 170 days composed the data collection period.

The feed intake of the grazing Datong yaks was determined, and concentrated feed was prepared based on the Chinese Beef Cattle Feeding Standard recommendations (NY/T 815-2004). The ingredient composition and nutrient levels of the diet given to the yaks in the experimental group are presented in Table 3. The roughage was a mixed ration of maize silage and oat hay (1:1). The daily diet of each yak contained 4 kg of concentrate and 2 kg of roughage (dry matter basis) during the experimental period. The mixed diet nutrient composition was determined in the Qinghai University laboratory according to the ‘Feed Analysis and Quality Test Technology’ methods^9^.

**Table 3:** Ingredients and proximate nutrition content of concentrate.

Composition	Content (%)	Nutritional composition	Content
Maize	67.0	ME b	3.01 Mcal/kg
Soybean meal	5.0	CP	14.88%DM
Cottonseed meal	10.0	Ca	0.66%DM
Wheat bran	14.0	P	0.43%DM
Salt	0.5		
Sodium Bicarbonate	1.5		
Stone Powder	1.5		
Premix Feed a	0.5		

^a^The premix provided the following per kg of diets: VA 12000 IU, VD 20 00 IU, VE 30 IU, Cu 12 mg, Fe 64 mg, Mn 56 mg, Zn 60 mg, I 1.2 mg, Se 0.4 mg, Co 0.4 mg;

^b^ME was calculation value (NRC, 2007), others were measured value.

### Measurement of samples

Datong male yaks were stunned with a captive bolt pistol, and the blood was drained at the end of sample collection. The jejunum was immediately isolated after slaughter, and the jejunal contents (10 g) were aliquoted into 2 mL sterile tubes and then stored at − 80 °C for digestive enzyme activity analysis. The activity of AMS, TRS, LPS and CLS was determined according to Fan’s method using commercially available kits from Takara^10^.

The harvested jejunal tissue was divided into two parts. One part was fixed in 4% buffered formaldehyde for 72 h. These samples were dehydrated under different concentrations of glucose and embedded in frozen embedding medium. Tissue sections (5–7 µm) were cut and stained with hematoxylin and eosin (HE) for analysis of villus height, villus width, crypt depth, mucosal thickness and muscular thickness. Images of each transverse section of jejunal tissue were acquired using DP70 software (Olympus, Nagano, Japan) and a BX51 microscopy (Olympus, Nagano, Japan). Measurements were made using Image Pro-Plus 5.1 software. Ten well-oriented and intact crypt-villus units on each slide were measured in triplicate^11^.

The other part of the jejunal tissue was aliquoted into a 1.5 mL centrifuge tube after removal of the surface contaminants via washing with phosphate-buffered saline solution and was stored in an ultracold storage freezer at − 80 °C until proteomics and RT-PCR analyses. The proteomics analysis was conducted by Novogene (China, Beijing). There were 3 replicates in each group. Jejunal proteins were extracted by the lysis buffer method^12^. Digestion of the protein (250 μg for each sample) was performed according to the FASP procedure. Label-free mass spectrometry (MS) experiments were performed on a Q Exactive mass spectrometer that was coupled to an Easy nLC system (Thermo Fisher Scientific, MA, USA). The MS data were analyzed using MaxQuant software and compared with the UniProt Bos taurus database. RT-PCR was performed to determine the copy numbers of target genes. The primers were synthesized at Shanghai Biological Engineering Ltd., China. RT-PCR was carried out in a LightCycler 480 Instrument (Fritz Hoffmann-La Roche Co. Ltd., Basel, Switzerland).

### Statistical analysis

The bioinformatics results were analyzed with an R language toolkit by Novogene (China, Beijing). Functional annotation and classification of all identified proteins were performed using the Blast2GO and InterProScan program with the Bos taurus UniProt database (uniprotbovin_170221.fasta). Pathway analyses were performed using the search pathway tool of the KEGG Mapper platform (http://www.genome.jp/kegg/) and the BLAST. Pathway enrichment was analyzed with Fisher's exact test, and pathways with corrected P values < 0.05 were defined as significantly enriched pathways. The Search Tool for the Retrieval of Interacting Genes/Proteins (STRING, http://string-db.org/) was used for prediction of physical and functional interactions in order to create the PPI. Graphical visualizations and interaction network analysis were performed in Cytoscape software.

The data are expressed as the mean ± standard deviation (SD). Duncan’s post hoc test was used to determine the significance of differences between the two groups. Differences were considered significant at P < 0.05 and extremely significant at P < 0.1.

### Ethics approval

All procedures involving the use of animals were approved by the Animal Care Committee of Lanzhou Institute of Animal Science and Veterinary Pharmaceutics, Chinese Academy of Agricultural Sciences, China (QHDX-18-04-07-01). The slaughter was conducted in accordance with the National Administration of Cattle Slaughtering and Quarantine Regulations (Qinghai, China).

## Supplementary Information

Supplementary information.

## Data Availability

The datasets generated during and/or analysed during the current study are available from the corresponding author on reasonable request.
