# Supplementary material for: Effect of high proportion concentrate dietary on Yak jejunal structure, physiological function and protein composition during cold season
Source: Sci Rep. 2021 Mar 9;11:5502. doi: 10.1038/s41598-021-84991-3 (PMC7970894; doi:10.1038/s41598-021-84991-3)
Supplement: Supplementary file 1 — Supplementary information. [file 41598_2021_84991_MOESM1_ESM.docx]

**Table S1** The list of differentially abundant proteins

|  | Protein name | No. NCBI | Data Sources | Molecular weight | PI | A.vs.B | P Value |
| --- | --- | --- | --- | --- | --- | --- | --- |
| 1 | Peroxisomal acyl-coenzyme A oxidase 1 | gi\|1542904629 | Bos taurus | 74648.73 | 8.64 | down | 0.017765 |
| 2 | Trifunctional enzyme subunit beta | gi\|528969156 | Bos taurus | 53704.13 | 9.47 | down | 0.005205 |
| 3 | Acetyl-CoA acetyltransferase | gi\|114050959 | Bos taurus | 44889.16 | 8.98 | down | 0.005205 |
| 4 | Acyl-CoA synthetase long chain family member 1 | gi\|115497270 | Bos taurus | 81442.47 | 6.53 | up | 0.021349 |
| 5 | Acyl-CoA synthetase long-chain family member 5 | gi\|115497154 | Bos taurus | 76028.39 | 7.99 | up | 0.034404 |
| 6 | Non-specific lipid-transfer protein | gi\|94966815 | Bos taurus | 42833.01 | 6.91 | down | 0.02393 |
| 7 | Calmodulin-dependent protein kinase kinase 1 | gi\|262073073 | Bos taurus | 16703.3 | 4.1 | down | 0.002814 |
| 8 | Aldo-keto reductase family 1 member B1 | gi\|426228047 | Bos taurus | 36291.6 | 5.95 | down | 0.00192 |
| 9 | Retinol saturase | gi\|1866868211 | Bos taurus | 66776.67 | 8.95 | down | 0.015575 |
| 10 | UDP-glucuronosyltransferase | gi\|157278895 | Bos taurus | 59603.54 | 8.67 | down | 0.013567 |
| 11 | Myosin regulatory light chain MRCL3 | gi\|223633898 | Bos taurus | 19851.5 | 4.72 | up | 0.017315 |
| 12 | Myosin light chain kinase | gi\|165875535 | Bos taurus | 16094.8 | 4.6 | up | 0.046408 |
| 13 | Retinol dehydrogenase 16 | gi\|47779226 | Bos taurus | 35668.54 | 9.14 | down | 0.004833 |
| 14 | UDP-glucose 6-dehydrogenase | gi\|27806365 | Bos taurus | 55136.32 | 7.51 | down | 0.032307 |
| 15 | Hexokinase | gi\|958310014 | Bos taurus | 102551.64 | 6.20 | down | 0.002719 |
| 16 | Protein S100-G | gi\|426216178 | Bos taurus | 8918.12 | 4.64 | down | 0.00381 |
| 17 | Copper transport protein ATOX1 | gi\|195539541 | Bos taurus | 7365.50 | 6.70 | down | 0.004881 |
| 18 | L-lactate dehydrogenase A chain | gi\|426252227 | Bos taurus | 112526 | 7.03 | down | 0.005694 |
| 19 | Potassium-transporting ATPase subunit beta-3 | gi\|78369424 | Bos taurus | 31521.22 | 6.74 | down | 0.03129 |
| 20 | Pyruvate kinase | gi\|1567495344 | Bos taurus | 56870.15 | 5.48 | down | 0.021687 |
| 21 | Argininosuccinate synthase | gi\|296482159 | Bos taurus | 46417.21 | 7.15 | down | 0.004438 |
| 22 | Carbamoyl-phosphate synthase 1 | gi\|300795597 | Bos taurus | 164740.45 | 6.28 | down | 0.023013 |
| 23 | Rho-related GTP-binding protein | gi\|803191482 | Bos taurus | 21927.32 | 8.76 | down | 0.021294 |
| 24 | Heat shock protein 10 | gi\|27805927 | Bos taurus | 10931.69 | 8.89 | down | 0.006352 |
| 25 | Occludin | gi\|129270156 | Bos taurus | 59198.05 | 5.94 | up | 0.007359 |
| 26 | Transforming protein RhoA | gi\|28603756 | Bos taurus | 21768.13 | 5.83 | down | 0.035028 |
| 27 | Chloride channel accessory 4 | gi\|74268230 | Bos taurus | 103419.65 | 5.07 | down | 0.033856 |
| 28 | Apolipoprotein A-I | gi\|146186566 | Bos taurus | 30276.35 | 5.71 | down | 0.020293 |
| 29 | Fatty acid-binding protein 2 | gi\|70778942 | Bos taurus | 15036.19 | 6.62 | down | 0.005327 |
| 30 | Apolipoprotein C-III | gi\|46359698 | Bos taurus | 10692.13 | 5.02 | down | 0.023864 |
| 31 | Long-chain-fatty-acid--CoA ligase 5 | gi\|115497154 | Bos taurus | 76028.39 | 7.99 | down | 0.007906 |
| 32 | Protein kinase C-like 2 | gi\|453392 | Bos taurus | 70649.96 | 8.14 | up | 0.042979 |
| 33 | Long-chain fatty acid transport protein 4 | gi\|115496984 | Bos taurus | 72199.32 | 8.58 | up | 0.021626 |
| 34 | Claudin-1 | gi\|148222888 | Bos taurus | 22864.97 | 8.40 | up | 0.017799 |
| 35 | Tight junction protein | gi\|525328953 | Bos taurus | 194741.52 | 6.18 | up | 0.004475 |
| 36 | G protein-coupled receptor 41 | gi\|223636308 | Bos taurus | 36817.37 | 8.95 | down | 0.006025 |
| 37 | Interleukin-6 | gi\|27806867 | Bos taurus | 23758.52 | 7.58 | down | 0.012888 |
| 38 | Interleukin-2 | gi\|25990390 | Bos taurus | 17627.60 | 6.14 | down | 0.015372 |
| 39 | C-domain 1 | gi\|145279649 | Bos taurus | 36145.70 | 5.30 | down | 0.020157 |
| 40 | C-C motif chemokine 5 | gi\|74353831 | Bos taurus | 10058.74 | 9.12 | down | 0.005879 |
| 41 | Ryanodine receptor 1 | gi\|958955250 | Bos taurus | 564093.03 | 5.25 | down | 0.04095 |
| 42 | 2-oxoglutarate dehydrogenase | gi\|158262612 | Bos taurus | 115808.05 | 6.28 | down | 0.040755 |
| 43 | Aconitate hydratase | gi\|90970312 | Bos taurus | 85359.36 | 7.87 | down | 0.031513 |
| 44 | Kelch-like protein 41 | gi\|1207821979 | Bos taurus | 68110.24 | 5.11 | down | 0.002088 |
| 45 | Pyruvate dehydrogenase E1 component subunit alpha | gi\|148300624 | Bos taurus | 43387.83 | 8.32 | down | 0.035996 |
| 46 | Dihydrolipoyllysine-residue acetyltransferase component of pyruvate dehydrogenase complex | gi\|296480288 | Bos taurus | 69066.86 | 8.38 | down | 0.034084 |
| 47 | NADH dehydrogenase [ubiquinone] flavoprotein 1 | gi\|40538780 | Bos taurus | 50651.85 | 8.37 | down | 0.045603 |
| 48 | Dihydrolipoyl dehydrogenase | gi\|329663954 | Bos taurus | 54187.21 | 7.59 | down | 0.045181 |
| 49 | CoA ligase [ADP-forming] subunit beta | gi\|296481810 | Bos taurus | 50146.07 | 6.37 | down | 0.00929 |
| 50 | Fumarate hydratase | gi\|115495377 | Bos taurus | 54691.11 | 9.06 | down | 0.032058 |
| 51 | Carnitine O-acetyltransferase | gi\|1207832637 | Bos taurus | 71128.99 | 8.39 | down | 0.048562 |
| 52 | Elongation factor Tu | gi\|27806367 | Bos taurus | 49398.26 | 6.72 | down | 0.036207 |
| 53 | Thioredoxin-dependent peroxide reductase | gi\|75948233 | Bos taurus | 28195.22 | 7.15 | down | 0.025592 |
| 54 | Isocitrate dehydrogenase [NAD] subunit beta | gi\|75773784 | Bos taurus | 39667.84 | 6.76 | down | 0.000144 |
| 55 | Enoyl-CoA hydratase | gi\|67944525 | Bos taurus | 31243.31 | 8.82 | down | 0.032061 |
| 56 | Isocitrate dehydrogenase [NAD] subunit alpha | gi\|958862660 | Bos taurus | 39667.84 | 6.76 | down | 0.017597 |
| 57 | Hydroxyacyl-Coenzyme A dehydrogenase | gi\|114052468 | Bos taurus | 34389.88 | 9.22 | down | 0.009729 |
| 58 | CoA ligase [ADP/GDP-forming] subunit alpha | gi\|296482469 | Bos taurus | 36166.76 | 9.41 | down | 0.040856 |
| 59 | Cytochrome c | gi\|114051487 | Bos taurus | 11703.54 | 9.52 | down | 0.041543 |
| 60 | Voltage-dependent anion-selective channel protein 2 | gi\|73586695 | Bos taurus | 31619.56 | 7.47 | down | 0.034663 |
| 61 | ATP synthase subunit O | gi\|27806307 | Bos taurus | 23319.57 | 9.90 | down | 0.000745 |
| 62 | Dihydrolipoyllysine-residue succinyltransferase component of 2-oxoglutarate dehydrogenase complex | gi\|116242688 | Bos taurus | 48972.53 | 9.10 | down | 0.024212 |
| 63 | Cytochrome c oxidase subunit 5A | gi\|81674705 | Bos taurus | 16735.16 | 6.42 | down | 0.022578 |
| 64 | Pyruvate dehydrogenase protein X component | gi\|115496095 | Bos taurus | 53885.93 | 8.64 | down | 0.043426 |
| 65 | Cytochrome c1 | gi\|83638779 | Bos taurus | 35296.75 | 9.14 | down | 0.018916 |
| 66 | Zeta-crystallin | gi\|114326274 | Bos taurus | 35382.80 | 8.28 | up | 0.004417 |
| 67 | Proteasome subunit alpha type-5 | gi\|62751982 | Bos taurus | 26411.03 | 4.74 | down | 0.019233 |
| 68 | 28S ribosomal protein S36 | gi\|1937760040 | Bos taurus | 11543.25 | 10.08 | down | 0.016864 |
| 69 | Ubiquinone biosynthesis protein COQ9 | gi\|958254576 | Bos taurus | 35778.47 | 5.56 | down | 0.043841 |
| 70 | Cytochrome c oxidase subunit 5B | gi\|151556362 | Bos taurus | 13833.80 | 8.80 | up | 0.002215 |
| 71 | Peptidyl-prolyl cis-trans isomerase A | gi\|75948300 | Bos taurus | 17869.35 | 8.34 | down | 0.037926 |
| 72 | Calcium voltage-gated channel auxiliary subunit alpha2delta 1 | gi\|329744564 | Bos taurus | 65748.56 | 6.33 | down | 0.027435 |
| 73 | Prohibitin | gi\|77736091 | Bos taurus | 29804.10 | 5.57 | up | 0.024844 |
| 74 | Complex I-B14 | gi\|28461207 | Bos taurus | 15053.49 | 10.04 | down | 0.00195 |
| 75 | Vesicle-associated membrane protein-associated protein A | gi\|115496338 | Bos taurus | 27856.35 | 8.80 | down | 0.013322 |
| 76 | ATP synthase subunit delta | gi\|28603800 | Bos taurus | 17612.08 | 5.15 | down | 0.007752 |
| 77 | Heterogeneous nuclear ribonucleoprotein K | gi\|77736071 | Bos taurus | 51019.22 | 5.14 | down | 0.032428 |
| 78 | Cytochrome c oxidase subunit 4 isoform 1 | gi\|74354123 | Bos taurus | 19571.59 | 9.32 | down | 0.009146 |
| 79 | Cytochrome b-c1 complex subunit 6 | gi\|77736435 | Bos taurus | 10623.73 | 4.45 | down | 0.015136 |
| 80 | Calumenin | gi\|77736275 | Bos taurus | 37082.69 | 4.41 | up | 0.013347 |
| 81 | NADH dehydrogenase [ubiquinone] 1 alpha subcomplex subunit 7 | gi\|28603786 | Bos taurus | 12676.59 | 10.06 | down | 0.039745 |
| 82 | 60S acidic ribosomal protein P2 | gi\|85812225 | Bos taurus | 11702.00 | 4.49 | down | 0.01511 |
| 83 | Cytochrome c oxidase subunit NDUFA4 | gi\|28461259 | Bos taurus | 9324.72 | 9.57 | down | 0.032895 |
| 84 | Cytochrome c oxidase subunit 6B1 | gi\|28603814 | Bos taurus | 10156.43 | 8.76 | down | 0.025536 |
| 85 | Dehydrogenase/reductase SDR family member 7B | gi\|1937741804 | Bos taurus | 35031.78 | 9.67 | down | 0.042274 |
| 86 | Adenylate kinase 4 | gi\|197097390 | Bos taurus | 25348.15 | 7.82 | up | 0.009294 |
| 87 | Phosphatidylinositol 4-kinase beta | gi\|73853758 | Bos taurus | 28211.74 | 4.81 | up | 0.01458 |
| 88 | Cytochrome b-c1 complex subunit 9 | gi\|165973984 | Bos taurus | 7457.63 | 9.16 | down | 0.046377 |
| 89 | NADH dehydrogenase [ubiquinone] 1 alpha subcomplex subunit 2 | gi\|28461257 | Bos taurus | 11079.81 | 9.93 | down | 0.007825 |
| 90 | Transgelin-2 | gi\|61888874 | Bos taurus | 22426.49 | 8.40 | up | 0.02854 |
| 91 | Succinate-semialdehyde dehydrogenase | gi\|1937738627 | Bos taurus | 56096.37 | 8.73 | down | 0.01205 |
| 92 | Neurofilament light polypeptide | gi\|1387217148 | Bos taurus | 62645.70 | 4.58 | down | 0.024395 |
| 93 | Peptidyl-prolyl cis-trans isomerase D | gi\|27806463 | Bos taurus | 40620.31 | 6.12 | down | 0.013514 |
| 94 | Thioredoxin like 1 | gi\|958195635 | Bos taurus | 32746.09 | 4.95 | down | 0.011477 |
| 95 | NADH dehydrogenase [ubiquinone] iron-sulfur protein 8 | gi\|705043959 | Bos taurus | 23896.37 | 6.44 | down | 0.017765 |
| 96 | Carnitine O-palmitoyltransferase 2 | gi\|114052655 | Bos taurus | 74482.77 | 8.36 | up | 0.005205 |

Note. A was grazing reared group; B was intensively reared group. PI was Isoelectric Point.
